# Phosphoribosylformylglycinamidine Synthase (PFAS) Deficiency: Clinical, Genetic and Metabolic Characterisation of a Novel Defect in Purine *de Novo* Synthesis

**DOI:** 10.1002/jimd.70041

**Published:** 2025-05-27

**Authors:** Marie Zikanova, Vaclava Skopova, Kyra E. Stuurman, Veronika Baresova, Olga Souckova, Ales Hnizda, Matyas Krijt, Anthony J. Bleyer, Jiri Zeman, Stanislav Kmoch

**Affiliations:** ^1^ Research Unit for Rare Diseases, Department of Paediatrics and Inherited Metabolic Disorders, First Faculty of Medicine Charles University in Prague and General University Hospital in Prague Czechia Czech Republic; ^2^ Department of Clinical Genetics Erasmus MC University Medical Center Rotterdam the Netherlands; ^3^ OMICS Mass Spectrometry Core Facility, Biology Departments, Faculty of Science Charles University, BIOCEV Vestec Czech Republic; ^4^ Section on Nephrology Wake Forest School of Medicine Winston‐Salem USA

**Keywords:** FGAR, formylglycinamide riboside, metabolic disorder, PFAS deficiency, purine *de novo* synthesis, purinosome

## Abstract

Purine de novo purine synthesis involves 10 reactions catalysed by six enzymes, including phosphoribosylformyglycinamidine synthase (PFAS). To date, genetic defects of three of these enzymes, namely ATIC, ADSL and PAICS, have been characterised in humans. Here, we report for the first time two individuals with PFAS deficiency. Probands were identified through metabolic and genetic screening of neurologically impaired individuals. The pathogenicity of the variants was established by structural and functional studies. Probands C1 and C2 presented with prematurity, short stature, recurrent seizures and mild neurological impairment. C1 had elevated urinary levels of formylglycineamide riboside (FGAr) and bi‐allelic PFAS variants encoding the NP_036525.1:p.Arg811Trp substitution and the NP_036525.1:p.Glu228_Ser230 in‐frame deletion. C2 is a 20‐year‐old female with a homozygous NP_036525.1:p.Asn264Lys substitution. These amino acid changes are predicted to affect the structural stability of PFAS. Accordingly, C1 skin fibroblasts showed decreased PFAS content and activity, with impaired purinosome formation that was restored by transfection with pTagBFP_PFAS_wt. The enzymatic activities of the corresponding recombinant mutant PFAS proteins were also reduced, and none of them, after transfection, corrected the elevated FGAR/r levels in PFAS‐deficient HeLa cells. While genetic defects in purine de novo synthesis are typically considered in patients with severe neurological impairment, these disorders, especially PFAS deficiency, should also be considered in milder phenotypes.

## 1 Introduction

**FIGURE 1:**
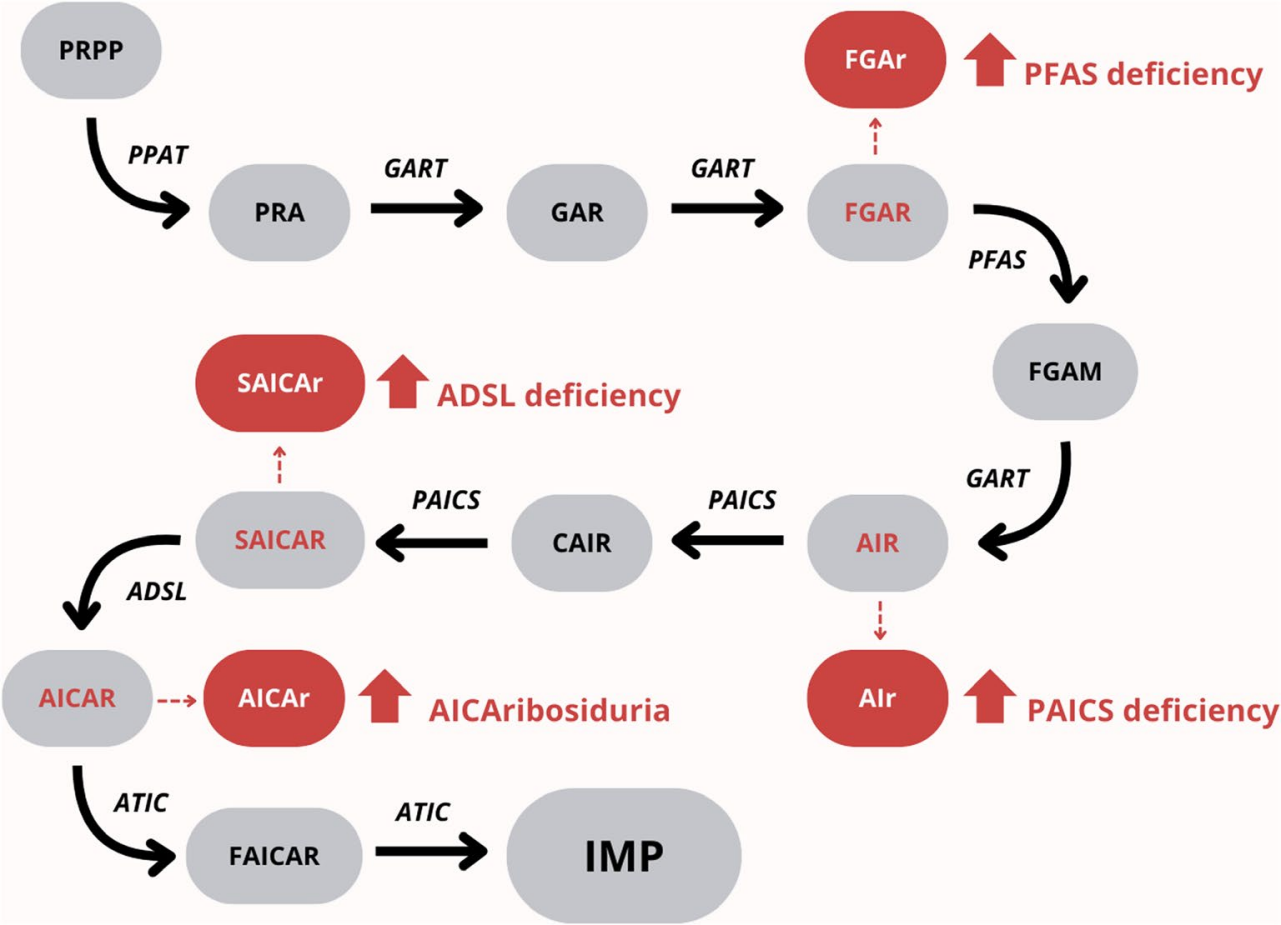
Purine *de novo* synthesis and its defects. Purine *de novo* synthesis consists of a series of 10 enzymatic reactions catalysed by six key enzymes: Phosphoribosylamidotransferase (PPAT), trifunctional phosphoribosylglycinamide formyltransferase/phosphoribosylglycinamide synthetase/phosphoribosylaminoimidazole synthetase (GART), phosphoribosylformylglycinamidine synthase (PFAS), bifunctional aminoimidazole ribonucleotide carboxylase/phosphoribosylaminoimidazolesuccinocarboxamide synthase (PAICS), adenylosuccinate lyase (ADSL) and bifunctional 5‐aminoimidazole‐4‐carboxamide ribonucleotide transformylase/IMP cyclohydrolase (ATIC). Enzyme deficiencies in this pathway are characterised biochemically by the accumulation of specific intermediates in patients' body fluids: PFAS deficiency leads to the accumulation of formylglycinamide riboside (FGAr); PAICS deficiency to aminoimidazole riboside (AIr); ADSL deficiency to succinylaminoimidazole carboxamide riboside (SAICAr) and succinyladenosine (SAdo) – the latter being an intermediate of the purine nucleotide cycle (not shown); and AICAribosiduria (ATIC deficiency) to the accumulation of 5‐aminoimidazole‐4‐carboxamide ribonucleoside (AICAr), SAICAr and SAdo.

Purine *de novo* synthesis comprises a series of 10 enzymatic reactions that are catalysed by six enzymes that, based upon cellular demand, temporarily assemble in the cytoplasm into liquid‐like condensates called purinosomes (Figure 1) [1, 2]. To date, genetic defects in three of these enzymes have been described. Adenylosuccinate lyase (ADSL, EC 4.3.2.2) deficiency [OMIM 103050] is characterised by the urinary and plasma accumulation of succinylaminoimidazole‐carboxamide riboside (SAICAr) and succinyladenosine (SAdo) [3]. Patients typically have severe neurologic manifestations, with some exceptions [4]. AICA‐ribosiduria [OMIM 608688] is characterised by urinary and plasma accumulation of 5‐aminoimidazole‐4‐carboxamide ribonucleoside (AICAr), SAICAr and SAdo, with clinical manifestations quite similar to ADSL deficiency [5, 6]. Aminoimidazole ribonucleotide carboxylase (AIRC, EC 4.1.1.21)/ phosphoribosylaminoimidazole succinocarboxamide synthase (SAICAIRS, EC 6.3.2.6) (PAICS) deficiency [OMIM 619859] is characterised by urinary and plasma accumulation of aminoimidazole riboside (AIr) and carboxyaminoimidazole riboside (CAIr), resulting in multiple developmental abnormalities [7, 8].

Here we report for the first time clinical, genetic and metabolic correlates of phosphoribosylformylglycineamidine synthase (PFAS) deficiency, which was identified in two individuals with prematurity, short stature and recurrent episodes of seizures with mild cognitive impairment.

## 2 Methods

Sequencing analysis, cultivation of primary skin fibroblasts (SF), lysate preparation, western blot analysis, LC–MS/MS analysis of urine, serum and fibroblast lysates, confocal microscopy, fluorescence image acquisition, analysis and transfection of PFAS‐deficient HeLa cells with pcDNA4 vectors have been performed as previously described [7, 8]. Detailed protocols for all methods are provided in the Appendix S1.

### 2.1 pTagBFP_PFAS and pcDNA4_Flag_PFAS Preparation

The eukaryotic *PFAS* gene, kindly provided by Stephen Benkovic from Penn State University, State College, Pennsylvania, United States, was cloned into the vectors pTagBFP‐C1 (Evrogen) and pcDNA4_mycHis (Invitrogen), respectively. In the latter vector, the Flag epitope was introduced at the 5' end of the *PFAS* gene. Subsequently, the pcDNA4_Flag_PFAS_Glu228‐Ser230del, pcDNA4_Flag_PFAS_Arg811Trp, pcDNA4_Flag_PFAS_Asn264Lys, pTagBFP_PFAS_Glu228‐Ser230del, pTagBFP_PFAS_Arg811Trp and pTagBFP_PFAS_Asn264Lys constructs were prepared by Site‐Directed Mutagenesis.

### 2.2 PFAS Enzyme Catalytic Activity

PFAS catalytic activity was measured in SF and recombinant protein samples. Recombinant Flag‐PFAS proteins were produced 24 h post‐transfection in HeLa PFAS deficient cells [9, 10], which were transiently transfected with expression vectors encoding Flag‐PFAS variants. The catalytic activity of PFAS was assayed in a coupled reaction with the recombinant protein MBP‐AIRS (5′‐phosphoribosylformylglycinamidine synthetase; EC 6.3.3.1) cloned from *E. coli* . MBP (maltose‐binding protein) was used as a fusion tag to facilitate protein expression and purification. The reaction used labeled ^13^C_2_‐formylglycinamide ribotide (FGAR) as substrate, and the product was measured by LC–MS/MS.

### 2.3 Structural Analysis

Structural analysis of PFAS mutants was performed with a theoretical model generated by Alpha‐Fold (AF‐O15067‐F1). The catalytic pocket with bound substrates was localised using structural alignment with the crystal structure of PFAS from *Salmonella typhimurium* (PDB ID 1T3T) and from *Thermotoga maritima* (PDB ID 2HS4). Structural models were analysed and visualised by PyMOL Viewer and UCSF ChimeraX.

## 3 Results

**FIGURE 2:**
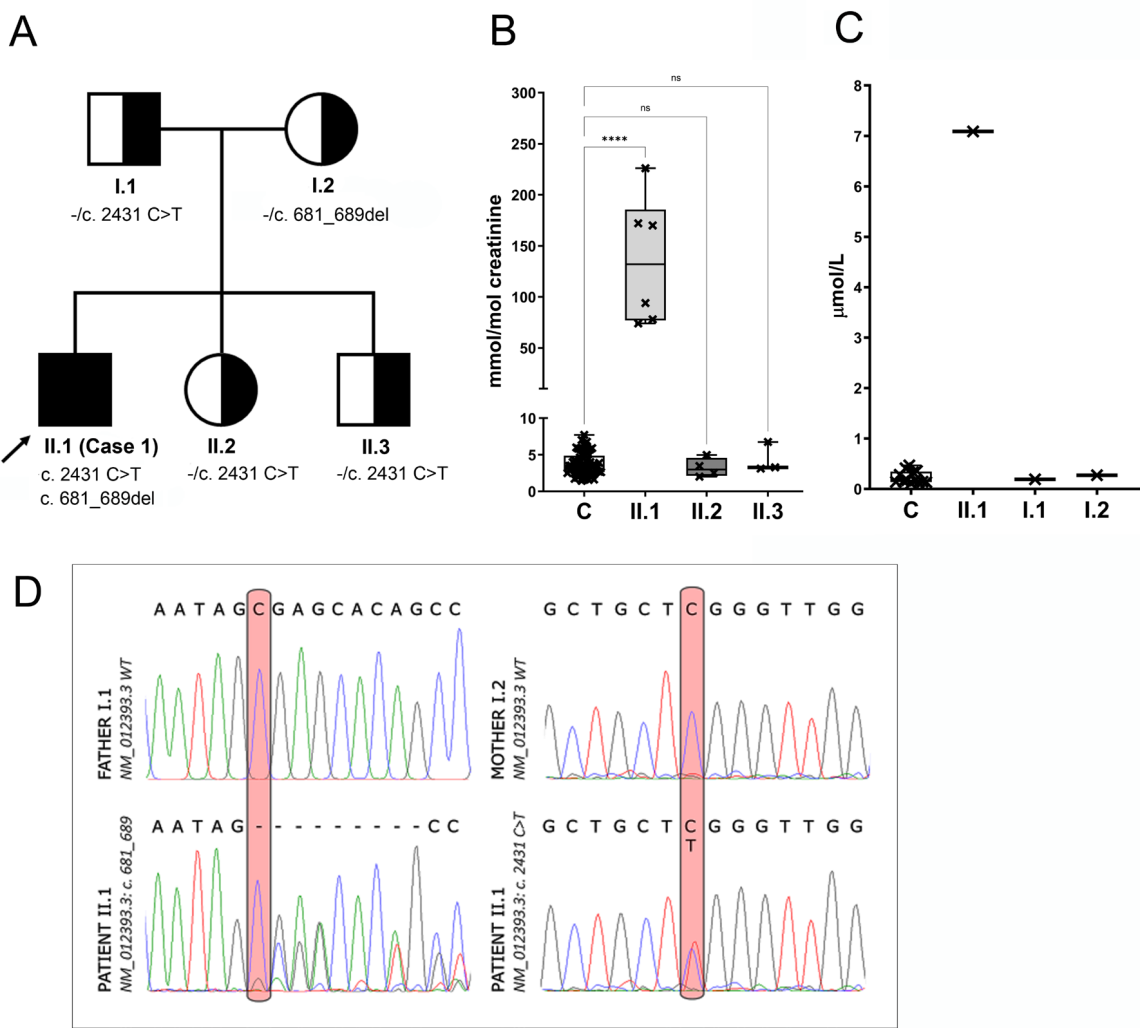
Pedigree, cDNA sequencing and LC–MS/MS profiling of purine metabolites in urine and serum samples from the case 1 family. (A) Pedigree of the family: Black symbols represent the affected individual, (+/−) indicates the presence (+) or absence (−) of the noted variant. (B) Accumulation of formylglycinamide riboside (FGAr) in the urine and (C) serum of the patient and his siblings or parents. FGAr concentrations were significantly increased in both the urine and serum of the patient compared to control samples. Data are presented in standardised boxplot graphs, showing the box from the first to third quartiles, whiskers representing the minimum and maximum, and all data points displayed. Statistical significance was assessed using one‐way ANOVA in GraphPad software, with *p*‐values indicated as **** for *p* ≤ 0.0001 and ns (non significant) for *p* > 0.05. (D) cDNA sequencing of PFAS transcripts from patient blood shows an in‐frame deletion (NM_012393.3:c.681_689del) caused by the genomic PFAS variant NC_000017.10:g.8159584G>A. This aberrant splicing is absent in the paternal cDNA, which shows only the wild‐type sequence. A second variant, NM_012393.3:c.2431C>T (missense), is present in the patient and the maternal cDNA displays only the wild‐type sequence at this site.

The first case (II.1 in Figure 2A) was identified from a biochemical screening of patients with various forms of neurological impairment [11]. The LC–MS/MS screening of purine *de novo* synthesis intermediates in urine samples revealed abnormally high urinary concentrations of formylglycinamide riboside (FGAr). Follow‐up biochemical studies conducted from 3 to 8.5 years of age revealed consistently elevated urinary concentrations of FGAr varying between 74 and 170 mmol/mol creatinine (normal range 1.6–6.9), with normal levels of other purine intermediates, including glycineamide riboside (GAr), formylglycineamidine riboside (FGAM), AIr, CAIr, SAICAr, AICAr and formylaminoimidazole carboxamide riboside (FAICAr), SAdo, inosine, xanthine, hypoxanthine and uric acid. Metabolic investigations conducted in the proband's clinically unaffected siblings (II.2 and II.3, respectively) revealed no abnormalities (Figure 2B). The serum FGAr concentration increased to 7 μmol/L (normal range 0.07–0.3) at 3 years of age, with no other abnormalities in purine intermediates. Metabolic investigations conducted in the proband's clinically unaffected parents (I.1 and I.2, resp.) revealed no abnormalities (Figure 2C).

The boy was born prematurely at 28 weeks of gestation with intrauterine growth retardation (IUGR), birth weight of 420 g and length of 28 cm. Early postnatal adaptation was uneventful with Apgar scores of 8 and 9 at 5 and 10 min, respectively. However, he required a prolonged stay in the neonatal intensive care unit due to bronchopulmonary dysplasia. Clinical investigations revealed a large patent ductus arteriosus and mild foramen ovale. During infancy, he had mild to moderate delays in the developmental milestones. He required surgical repair for hypospadias. At 4 years, he had two epileptic seizures, characterised as benign epilepsy with occipital spikes (BEOS—Panayitopoulose variant). At the current age of 8.5 years, he is 125.5 cm tall (5th percentile), weighs 22 kg (5th percentile) and has a head circumference of 49 cm (< 3rd percentile). Clinically, he has attention deficit hyperactivity disorder and anxiety depressive disorder. Electroencephalography revealed epileptic graphoelements with sharp spikes and slow waves in the right parietal and left occipital regions. Both parents and two siblings are healthy.

**FIGURE 3:**
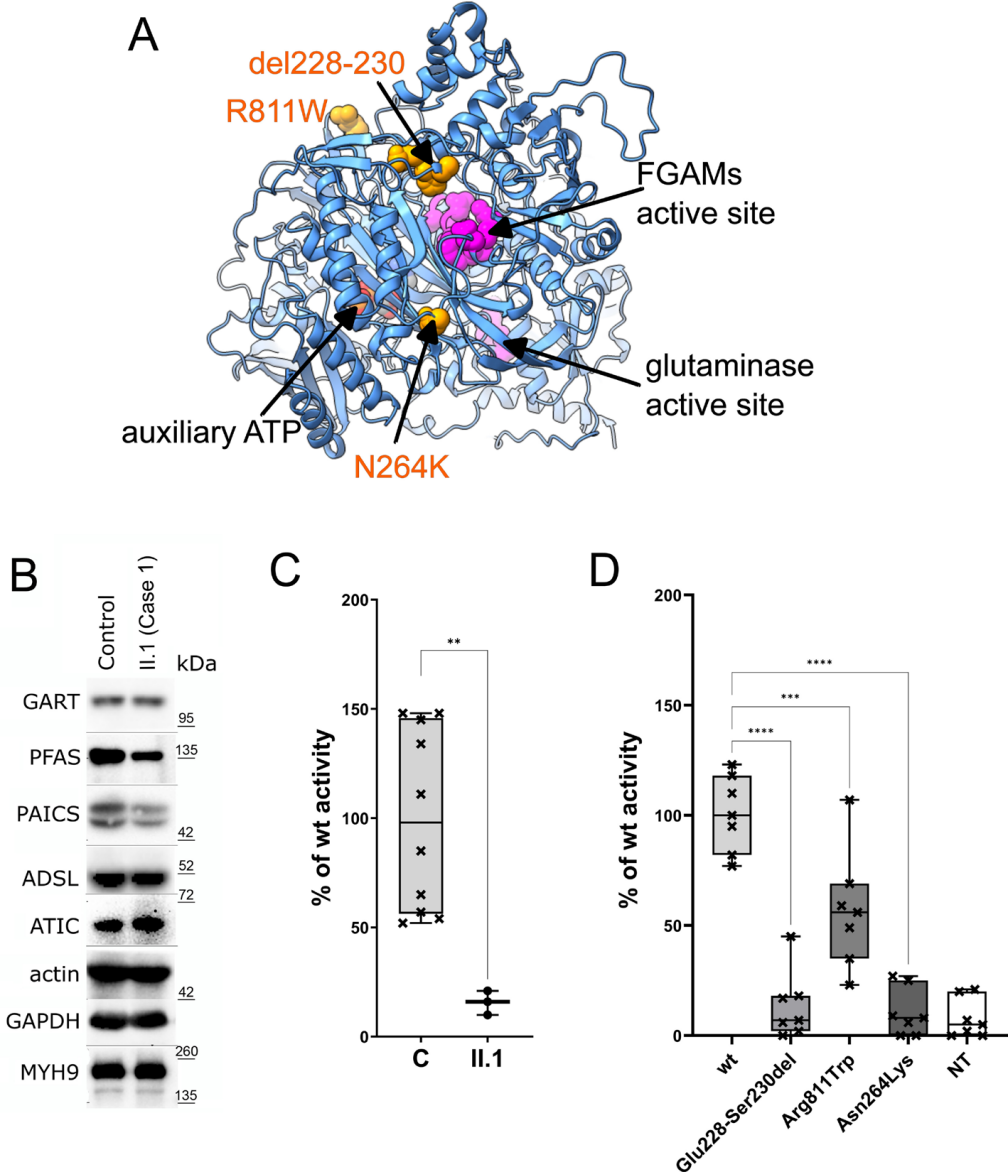
Characterization of PFAS variants: Structural and functional analyses in fibroblasts and recombinant proteins. (A) Structural mapping of the variants using a theoretical model generated by Alpha‐Fold (AF‐O15067‐F1). The positions of the auxiliary ATP and active sites were localised based on the crystal structures of PFAS from *Salmonella typhimurium* (PDB ID: 1T3T) and from *Thermotoga maritima* (PDB ID: 2HS4). The positions of the variants are highlighted as orange spheres. The in‐frame deletion Glu228_Ser230del affects a flexible part of the helical region connecting the linker between the N‐domain and the FGAM synthetase domain. The Asn264Lys and Arg811Trp substitutions are located at the FGAM synthetase domain, distant from the active site. They can induce local destabilization due to steric clashes and gain of polar interaction with Asp761 in the case of Asn264Lys or due to the presence of a more hydrophobic residue at the protein surface in the case of Arg811Trp. (B) Western blot analysis of skin fibroblasts using antibodies against GART, PFAS, PAICS, ADSL and ATIC revealed reduced levels of PFAS and PAICS, while the levels of other DNPS enzymes were comparable to those in control fibroblasts. Protein levels were normalised to GAPDH, Actin and MYH9. (C) PFAS enzyme activity in fibroblasts from case 1 was reduced to 16% of control levels. (D) The catalytic activity of the recombinant Flag_PFAS protein Glu228‐Ser230del was decreased to 14%, Arg811Trp to 57% and Asn264Lys to 11% activity compared to Flag_PFAS_wt. Data are presented as standardised boxplot graphs, with the box spanning the first to third quartiles, whiskers showing the minimum and maximum values, individual data points displayed, and the median represented by a line. Experiments were performed at least three times (*n* ≥ 3). Statistical significance was assessed using one‐way ANOVA in GraphPad software, with *p*‐values indicated as ** for *p* ≤ 0.01, *** for *p* ≤ 0.001 and **** for *p* ≤ 0.0001.

**FIGURE 4:**
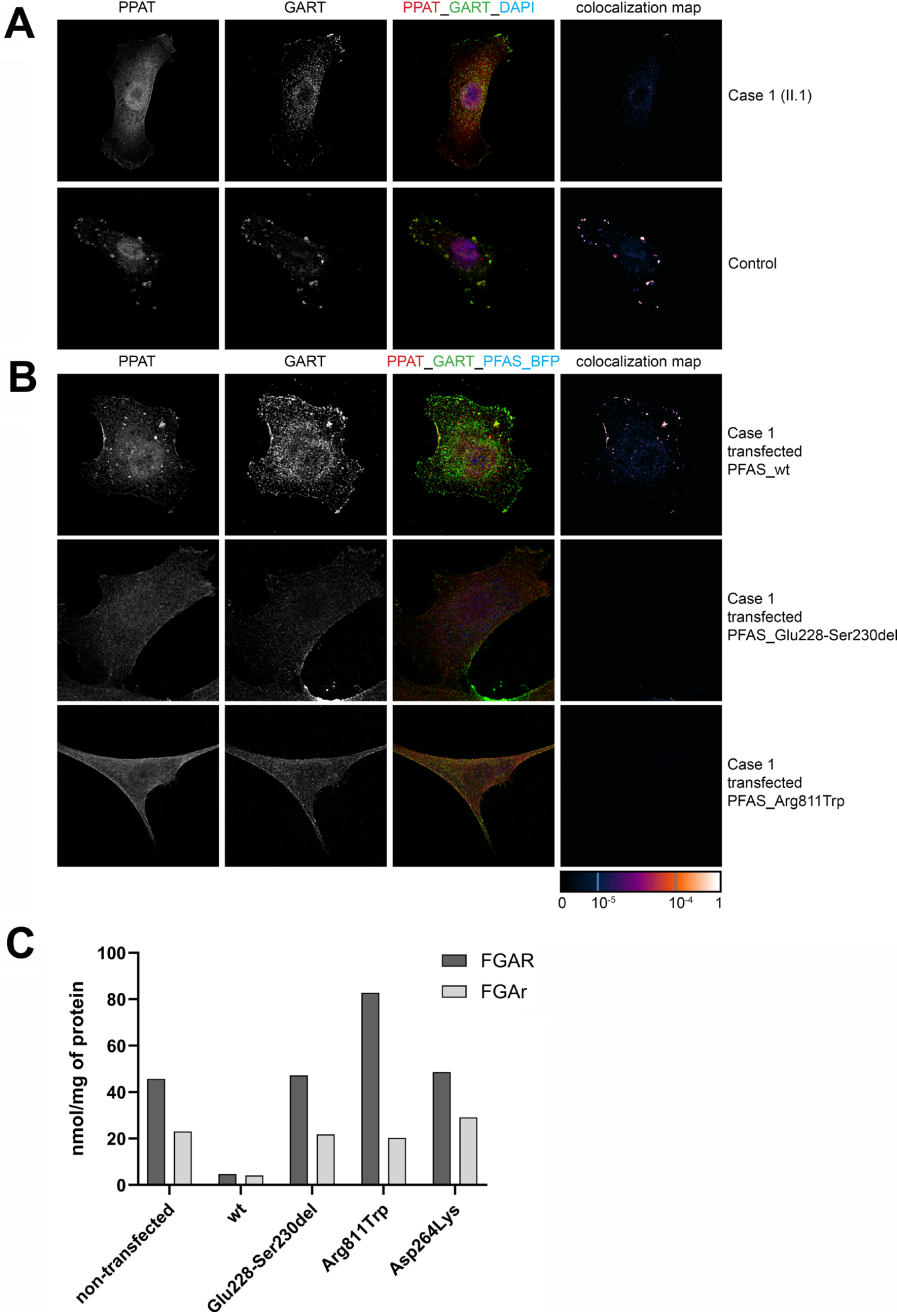
Functional complementation of PFAS deficiency. (A) Purinosome formation in skin fibroblasts of case 1. Patient and control skin fibroblasts were cultured in purine‐depleted medium and confocal fluorescence microscopy was used to detect fluorescently labeled endogenous proteins PPAT and GART. In control fibroblasts, purinosome formation was observed, characterised by granular staining and significant colocalization of PPAT and GART in the cytoplasm. In contrast, fibroblasts from case 1 displayed diffuse cytosolic staining of PPAT and GART with no significant overlapping signals. (B) Purinosome formation was restored following transient transfection with an eukaryotic expression vector encoding wild‐type PFAS (pTagBFP_PFAS_wt). However, transfection with vectors encoding pTagBFP_PFAS variants, Glu228‐Ser230del and Arg811Trp, failed to restore purinosome formation. (C) Transfection of PFAS‐deficient HeLa cells with the pcDNA4_Flag_PFAS_Glu228‐Ser230del, pcDNA4_Flag_PFAS_Arg811Trp and pcDNA4_Flag_PFAS_Asn264Lys constructs corresponding to patients' variants did not correct FGAR/r levels in cell lysate, whereas transfection with the wild‐type construct decreased both.

Since elevated urinary and serum concentrations of FGAr suggested PFAS deficiency, targeted sequencing of *PFAS* complementary DNA and genomic coding regions was performed and revealed two variants: NC_000017.10:g.8159584G>A and g.8168756C>T (GRCh37). The first variant causes alternative splicing between intron 6 and exon 7, resulting in the in‐frame deletion NM_012393.3:c.681_689del, NP_036525.1:p.(Glu228_Ser230del) (Figure 2D). The variant is not reported in the Genome Aggregation Database (gnomAD) v2.1.1 and has been evaluated according to the ACMG guidelines as pathogenic (PP1, PS3, PM2, PM2). The second variant is a missense substitution, NM_012393.3:c.2431C>T, NP_036525.1:p.(Arg811Trp) (Figure 2D). This variant has no reported homozygous allele in both the gnomAD v2.1.1 genomes and exomes and has been evaluated according to the ACMG guidelines as likely pathogenic (PP1, PS3, PM3, PM2). Genotyping and segregation analysis in the family identified that the proband's parents and his biochemically and clinically unaffected siblings are heterozygous for these variants (Figure 2A). Structural modelling using the theoretical model generated by Alpha‐Fold (AF‐O15067‐F1) and the crystal structures of the catalytic pockets from *Salmonella typhimurium* (PDB ID 1T3T) and from *Thermotoga maritima* (PDB ID 2HS4) suggested that both amino acid changes potentially affect the structural stability of PFAS (Figure 3A). Consistently, western blot analysis and enzyme activity studies revealed that patients´ skin fibroblasts had about 30% PFAS protein levels (Figure 3B) and 16% PFAS enzyme activity (Figure 3C) compared to control fibroblasts. Enzymatic activities of the recombinant p.Arg811Trp and p.Glu228_Ser230del forms were reduced to 57% and 14% of the wild‐type PFAS, respectively (Figure 3D). Immunofluorescence and confocal microscopy studies demonstrated that when cultured in purine‐depleted media, the affected skin fibroblasts have a reduced ability to form purinosomes (Figure 4A). This was corrected by transfection with the pTagBFP_PFAS_wt but not the mutated constructs (Figure 4B). Consistently, transfection with the corresponding mutated pcDNA4_Flag_PFAS did not correct FGAR/r levels in PFAS deficient HeLa cell lysates whereas transfection with the wild‐type construct decreased both (Figure 4C) [9].

The second case was identified in the Netherlands through a collaboration facilitated by GeneMatcher (https://genematcher.org/) [12]. The proband presented to a clinical geneticist in her twenties. While she could not provide extensive details about her birth and early years, she recalled being small for gestational age with a low birth weight. Despite achieving normal developmental milestones, she remains short. Her height is 160 cm, which is −0.5 SDS on the World Health Organisation growth chart. She did not finish high school due to learning problems. She had her first seizure at age 20, when she fainted and vomited. She was found by her brother, who knew the symptoms from another sister who had epileptic seizures. There were no previous triggers, and she had never experienced this in the past, neither absences nor other forms of seizures. She does not drink alcohol, take drugs, or smoke. She did not seek medical evaluation at that time. Several years later, she had another seizure. Antiepileptic drugs were administered: first valproic acid and then lamotrigine. However, she stopped taking lamotrigine on her own and has never had a seizure since. Her medical history includes several bone fractures and joint dislocations. Physically, she is a slender woman with hirsutism on her cheekbones and upward‐slanting eyes. She is from a Turkish consanguineous family of 15 children, with six of her siblings also experiencing seizures. A family history of seizures prompted exome sequencing analysis, revealing that the proband carries the homozygous *PFAS* variant NM_012393.3:c.792C>G encoding the missense NP_036525.1:p.(Asn264Lys) substitution. This variant has not been reported in gnomAD v2.1.1 and has been evaluated according to the ACMG guidelines as likely pathogenic (PS3, PM3, PM2). The proband declined further examinations, with no biological material available for metabolic and enzymology investigations. Structural modelling revealed that the amino acid substitution potentially affects the structural stability of the PFAS (Figure 3A) and the enzymatic activity of the corresponding recombinant protein was reduced to 11% of the wild‐type PFAS (Figure 3D). Transfection of PFAS‐deficient HeLa cells with the pcDNA4_Flag_PFAS_Asn264Lys construct did not correct FGAR/r levels (Figure 4C).

## 4 Discussion

Purine *de novo* synthesis comprises a series of 10 enzymatic reactions that are catalysed by six enzymes. To date, genetic defects of three of these enzymes, namely ADSL, bifunctional 5‐aminoimidazole‐4‐carboxamide ribonucleotide transformylase/IMP cyclohydrolase (ATIC), and PAICS, have been characterised in humans. In this study, we report for the first time clinical, genetic and metabolic correlates of PFAS deficiency.

PFAS catalyses the fourth step of purine *de novo* synthesis and is a component of the protein core of the purinosome (Figure 1.) [13] The vital importance of purine *de novo* synthesis is demonstrated by the embryonic lethality caused by either pharmacologic inhibition [14] or loss‐of‐function mutations of the enzymes along the pathway, including phosphoribosylamidotransferase [15], trifunctional phosphoribosylglycinamide formyltransferase, phosphoribosylglycinamide synthetase, phosphoribosylaminoimidazole synthetase (GART) [16, 17], PAICS [7, 8, 16], ADSL [4, 18, 19] and ATIC [5, 6, 20, 21] in model organisms and humans.

Dominant PFAS mutations have been reported in mice with craniofacial abnormalities [22]. Recessive loss‐of‐function deletion of PFAS caused pupal lethality in *Drosophila melanogaster* [23]. The recessive missense form p.Arg1205Cys of PFAS has been identified as likely causal for embryonic lethality in cattle [24]. The p.Arg1205Cys amino acid substitution potentially affects the structural stability of PFAS by disrupting three polar interactions localised in the contact area between glutaminase and FGAMs domains.

The two probands reported in this investigation have bi‐allelic hypomorphic missense or in‐frame deleted *PFAS* variants that affect structurally important amino acid residues and decrease but do not eliminate PFAS protein amount and enzyme activity. Similar to other defects of enzymes of the purine *de novo* synthesis pathway [2, 7, 25], decreased PFAS content compromises purinosome formation and function in affected skin fibroblasts and potentially in other cell types. This leads to the accumulation of the PFAS substrate FGAR and of its dephosphorylated form FGAr, which are detectable in serum and urine. The pathogenicity of *de novo* purine synthesis deficiencies has been attributed to the cytotoxic effects of accumulated metabolites [4, 6]. Both FGAR and FGAr have demonstrated cytotoxic and neurotoxic effects on the viability of several cell lines, including a neuronal CAD‐2A2D5 (CAD5) cells [26]. Accordingly, probands presented with prematurity, short stature and recurrent seizures with relatively mild neurological manifestations. Their clinical presentation points to pathogenetic effects on purine metabolism and purinergic signalling in the musculoskeletal [27] and nervous systems [28, 29].

Genetic defects of purine *de novo* synthesis are rare. Estimates from loss‐of‐function allele frequencies reported in Exome Aggregation Consortium (ExAC) database suggest a prevalence of ADSL deficiency of approximately 1 in 1,240 ,710, with a carrier frequency of 1 in 557 [30]. A similar analysis using the gnomAD database identified 245 loss‐of‐function *PFAS* variants in 936 of 1609350 alleles and 30 ‘likely deleterious’ missense variants (defined by the Combined Annotation Dependent Depletion (CADD) score above 30) [31] in 1239 of 1,609,901 alleles. This suggests a combined carrier frequency of 1 in 722, with an estimated prevalence of PFAS deficiency of approximately 1 in 2,085,881, which is similar to ADSL deficiency. Genetic defects of purine *de novo* synthesis are usually considered in individuals with severe neurologic affection. Thus, the rarity of PFAS deficiency may be due to embryonic lethality related to loss‐of‐function *PFAS* variants and poor clinical recognition of phenotypically mild cases represented by the probands with hypomorphic *PFAS* variants reported here. To this end, we had the unique opportunity to characterise, for the first time, the presence of the purine *de novo* synthesis intermediates in clinical specimens from the proband with “mild” PFAS deficiency. In agreement with our cellular model of PFAS deficiency [9] we demonstrated in the proband constantly increased urinary and serum concentrations of FGAr; thus, this metabolite represents a specific diagnostic marker of PFAS deficiency.

In conclusion, we have identified a new inherited metabolic disorder of purine *de novo* synthesis: PFAS deficiency. The previous lack of detection of this condition may be related to the embryonic lethality of certain *PFAS* variants, poor clinical recognition of phenotypically mild cases, and limited access to specialised laboratory tests for purine intermediates.

## Author Contributions

M.Z. and S.K. wrote the manuscript text and prepared the figures. V.S., K.E.S. and O.S. performed the genetic analyses. J.Z., K.E.S. and A.J.B. collected clinical data and performed clinical data analyses. V.B. prepared cellular models and performed all the cell culture and microscopy experiments as well as microscopy data analysis. V.S., O.S., A.H., M.K. and M.Z. conducted functional studies. S.K., A.J.B. and M.Z. reviewed and edited the manuscript. All authors reviewed and approved the final version of the manuscript.

## Ethics Statement

All procedures followed were in accordance with the ethical standards of the responsible committee on human experimentation (institutional and national) and with the Helsinki Declaration of 1975, as revised in 2000 (5). Informed consent was obtained from all patients for being included in the study.

## Conflicts of Interest

The authors declare no conflicts of interest.

## Supporting information


Data S1.
