# Supplementary material for: Phosphoribosylformylglycinamidine Synthase (PFAS) Deficiency: Clinical, Genetic and Metabolic Characterisation of a Novel Defect in Purine de Novo Synthesis
Source: J Inherit Metab Dis. 2025 May 27;48(3):e70041. doi: 10.1002/jimd.70041 (PMC12107509; doi:10.1002/jimd.70041)
Supplement: Supplementary file 1 — Data S1. [file JIMD-48-0-s001.docx]

# Supplementary Appendix

# Methods

## Chemicals

^13^C_2_-FGAR, the substrate for PFAS activity assays, and all needed commercially unavailable standards for HPLC and LC-MS/MS were produced according to established methods.^1-3^ AICAR, IMP, inosine, hypoxanthine, xanthine, uric acid, ^15^N_2_-uric acid were purchased from Sigma-Aldrich, FAICAR, ^13^C_2_^15^N-AICAr from Toronto Research Chemicals, ^13^C_5_-hypoxanthine from Cambridge Isotope Laboratories, and CAIR from MedChemExpress, respectively. Sigma-Aldrich supplied the remaining reagents, except where noted differently.

## Sequencing analysis

Case 1: Genomic DNA and total RNA were isolated from venous blood following standard protocols. cDNA was synthesized using the ProtoScript II First Strand cDNA Synthesis Kit (NEB). PCR was performed in a 25 µl reaction mixture containing Red PCR Master Mix (Rovalab) and specific primers following a standard procedure. The amplicons were purified and sequenced using specific primers by Sanger sequencing (Eurofins Genomics).

Case 2: After enriching DNA using the Agilent Sureselect Clinical Research Exome (CRE) Capture, the samples were processed on the Illumina HiSeq platform. The objective was to achieve 50 million total reads per exome, with a mapped fraction greater than 0.98. The average coverage reached approximately 50x. The data were demultiplexed using Illumina's bcl2fastq software. Reads were mapped to the genome with BWA software. Variant detection was conducted using the Genome Analysis Toolkit (GATK), available at Broad Institute. The analysis was performed in Cartagenia on the HiSeq 4000 platform (150bp paired-end) with a focus on filtering for regions of homozygosity (ROH) variants. The variants were not confirmed through Sanger sequencing.

## pTagBFP_PFAS and pcDNA4_Flag_PFAS preparation

The eukaryotic PFAS gene, graciously provided by Stephen Benkovic from Penn State University, State College, Pennsylvania, United States, was cloned into the vectors pTagBFP-C1 (Evrogen) and pcDNA4_mycHis (Invitrogen), respectively. To the latter vector, the Flag epitope was introduced at the 5´end of *PFAS* gene using the NEBuilder HiFi DNA Assembly Kit (NEB). The Q5 Site-Directed Mutagenesis Kit (NEB) was subsequently used to induce the mutations c.681_689delCGAGCACAG, c.2431C>T, and c.792C>A, respectively and expression vectors: pcDNA4_Flag_PFAS_wt, pcDNA4_Flag_PFAS_Glu228-Ser230del, pcDNA4_Flag_PFAS_Arg811Trp, pcDNA4_Flag_PFAS_Asn264Lys and pTagBFP_PFAS_wt, pTagBFP_PFAS_Glu228-Ser230del pTagBFP_PFAS_Arg811Trp, pTagBFP_PFAS_ Asn264Lys were prepared. All constructs were verified by Sanger sequencing (Eurofins Genomics).

## Cultivation of Primary Skin Fibroblasts

Skin fibroblasts (SF) from Case 1 were maintained in the DMEM/F12 nutrition mix medium (Gibco, Invitrogen), supplemented with 10% fetal bovine serum (FBS; Gibco, Invitrogen) and 1% of penicillin/streptomycin (P/S, Sigma Aldrich) and 0.03 mM adenine. For detection of purinosome formation and LC-MS/MS analysis of DNPS metabolites, the cells were cultivated in the purine-depleted medium: Dulbecco’s minimum essential medium (DMEM, Gibco, Invitrogen) supplemented with 10% dialyzed FBS (dFBS)^4^ and 1% P/S (Sigma-Aldrich), 48 h prior the experiment.

## Cultivation of PFAS deficient HeLa cells

PFAS deficient HeLa cells were maintained in the DMEM/F12 nutrition mix medium (Gibco, Invitrogen), supplemented with 10% fetal bovine serum (FBS; Gibco, Invitrogen) and 1% of penicillin/streptomycin (P/S, Sigma Aldrich) and 0.03 mM adenine. For detection of LC-MS/MS analysis of DNPS metabolites, the cells were transfected (see below) and cultivated in the purine-depleted medium: Dulbecco’s minimum essential medium (DMEM, Gibco, Invitrogen) supplemented with 10% dialyzed FBS (dFBS)^4^, 24 h prior the experiment.

## Cell transfection

For immunofluorescence 1 × 10^4^ of patient or control skin fibroblasts were transiently transfected with 1.5 μg of pTagBFP_PFAS_wt, pTagBFP_PFAS_Glu228-Ser230del or pTagBFP_PFAS_Arg811Trp, using Neon™ Transfection System (Thermo Fisher), with 10 μl transfection tip, pulse voltage 1700 V, pulse width 20 ms, number of pulses 1.

For the PFAS enzyme catalytic activity 5 x 10^5^ of PFAS deficient HeLa cells were transiently transfected with 15 μg of pcDNA4_Flag_PFAS_wt, pcDNA4_Flag_PFAS_Glu228-Ser230del, pcDNA4_Flag_PFAS_Arg811Trp or pcDNA4_Flag_PFAS_Asn264Lys, using Neon™ Transfection System, with 100 μl transfection tip, pulse voltage 1005 V, pulse width 35 ms, number of pulses 2. Cells were seeded in purine depleted medium and analyzed 24 h post transfection.

## Lysates Preparation

1.5×10^6^ of SF cells were PBS-rinsed, centrifuged at 400x*g* for 5 minutes at 4°C, resuspended in 30 μl lysis mix (30 mM KH_2_PO_4_, pH 6.0, 0.5% polyethylene glycol ether W-1, Protease Inhibitor Cocktail Tablets), and iced for 45 minutes. The sample was sonicated (4x5s bursts), and centrifuged at 17000x*g* for 20 min at 4°C.

HeLa cells were washed with PBS and final pellets of the cells were dissolved in lysis buffer: 10 mM Tris pH 8.2, 2 mM EDTA, 10 mM KCl, 1 mM DTT, and 4% glycerol with Protease Inhibitor Cocktail Tablets (Roche) (for each 1 × 10^6^ cells/ 50 µl of buffer was used), sonicated four times for 15s and centrifuged at 17 000g for 20 min at 4°C. Final protein concentration in cell lysates was measured by Bradford method.

## LC-MS/MS analysis of urine, serum and skin fibroblast lysates

3x volume of frozen 80% methanol was added to 50 µl of serum, SF lysate with protein concentration adjusted to 1 mg/ml or cultivation media of SF. Samples were incubated ON at -80°C. The following day, the samples were centrifuged, and the supernatants were evaporated by speed-vac. Obtained pellets were resolved in 50 µl of water and vortexed. Urine samples were diluted to 0.1 mmol/l creatinine. All samples were centrifuged briefly and 45 µl of supernatant was mixed with 5 µl of internal labeled standard mixture containing ^13^C_2_-GAr, ^13^C_2_-FGAr, ^13^C_4_-SAICAr, ^13^C_2_^15^N-AICAr, ^13^C_4_-Sado, ^15^N_2_-uric acid, and ^13^C_5_-hypoxanthine. 5 µl of the sample was injected into the LC-MS/MS system consisting of an Agilent 1290 Infinity LC System (Agilent Technologies) equipped with a Prontosil 120-3-C18 AQ column (150*3 mm, 3 µm, Bischoff) coupled to an API 4000 triple quadrupole mass spectrometer with an electron spray ionization operated by Analyst software (Applied Biosystems), as previously described ^5, 6^. Selective reaction monitoring (SRM) was used for detection and quantification of selected compounds.^7^

## PFAS Enzyme Catalytic Activity

PFAS catalytic activity was measured in skin fibroblasts and recombinant proteins. The assay was coupled with MBP-AIRS reaction, and the intermediate phosphoribosylformylglycinamidine (FGAM) and the final product aminoimidazole ribotide (AIR) were measured. cDNA of the bacterial gene *AIRS* for AIR synthetase (EC 6.3.3.1) was cloned into the pMAL-C2 plasmid and the fusion protein MBP-AIRS was produced in *E.coli* and purified by affinity chromatography according to a standard protocol (NEB). The catalytic activity of PFAS in skin fibroblasts was assayed at 37°C, pH 7.5 in a coupled reaction with 0.4 µg/µl MBP-AIRS, 40 mM Tris, pH 8.0; 2 mM MgCl_2_, 2 mM glutamine, 2 mM ATP, 2 mM KCl, 0.4 µg/µl skin fibroblasts lysate and 150 µM ^13^C_2_-FGAR for 90 min. The reaction was stopped with 3 mM EDTA and deep frozen.

Recombinant Flag-PFAS proteins were produced 24h post transfection in HeLa PFAS KO cells ^8, 9^ transiently transfected with expression vectors with Flag_PFAS variants. The non-transfected control was also performed. Lysates were analyzed by Western blot using mouse monoclonal anti-Flag (Sigma, F1804) and rabbit polyclonal anti-PFAS antibodies (Aviva, ARP46181) and PFAS concentration was normalized using Image Lab Software (Bio-Rad). Then, the catalytic activity of recombinant PFAS proteins was assayed at 37°C, in a coupled reaction with 0.25 µg/µl MBP-AIRS, 40 mM Tris, pH 8.0; 2 mM MgCl_2_, 1 mM glutamine, 1.3 mM ATP, 40 mM KCl, 0.2 µg/µl Flag_PFAS lysate and 60 µM ^13^C_2_-FGAR for 20 min.

The samples were diluted (10 times for reactions with SF and 5 times for reactions with recombinant proteins, respectively) and injected into the LC-MS/MS according to the settings described above. Selective reaction monitoring (SRM) was used for quantification of the final product, ^13^C_2_-AIR.^1, 3^

## Western Blot Analysis

Skin fibroblast lysates underwent 10% SDS-PAGE separation, and proteins were transferred to a PVDF membrane. The membrane was blocked using 5% BSA in PBS-T (PBS with 0.1% Tween-20) and incubated with primary antibodies: anti-GART mouse monoclonal (H00002618-B01P, Abnova), anti-PFAS rabbit polyclonal (ARP46181, Aviva), anti-PAICS mouse monoclonal (TA501470, Origene), anti-ADSL rabbit polyclonal (HPA000525, Sigma), anti-ATIC mouse monoclonal (ab33520, Abcam), anti-GAPDH mouse IgM monoclonal (G8795, Sigma), anti-MYH9 mouse monoclonal (H00004627-M03, Abnova) or anti-actin polyclonal rabbit (A2103, Sigma) in 5% BSA in PBS-T. Peroxidase-conjugated secondary antibodies (goat anti-mouse IgG (Sigma) and IgM (Pierce) and goat anti-rabbit IgG (ThermoScientific), respectively) were used for detection. Chemiluminescence was achieved using Clarity or Clarity MAX Western ECL Substrate (Bio-Rad) and captured by ChemiDoc MP Imaging System (Bio-Rad).

## Structural Analysis

Structural analysis of PFAS mutants was performed with a theoretical model generated by Alpha-Fold (AF-O15067-F1). The catalytic pocket alongside with ATP was localized using structural alignment with crystal structure of PFAS from *Salmonella typhimurium* (PDB ID 1T3T) and from *Thermotoga maritima* (PDB ID 2HS4). Structural models were analyzed and visualized by Pymol Viewer and UCSF Chimera X.

## Immunofluorescence

For parallel immunodetection of Phosphoribosyl Pyrophosphate Amidotransformylaserase (PPAT); and Glycinamide Ribonucleotide Synthetase, Glycinamide Ribonucleotide Transformylase, and Aminoimidazole Ribonucleotide Synthetase (trifunctional GART) were the cells incubated with specific antibodies. PPAT was detected with polyclonal rabbit anti-PPAT antibody (ARP46079, Aviva), diluted 1:50, GART was detected with monoclonal mouse GART antibody (H00002618-B01P, Abnova) diluted 1:50, and incubated overnight at 4°C in humidified chamber.

Detection of bound primary antibodies was achieved using Donkey anti-Mouse IgG Alexa Fluor^®^488 and Goat anti-Rabbit IgG Alexa Fluor^®^555 secondary antibodies, (Thermo Fischer Scientific) diluted 1:1000 in 5% BSA in PBS incubated 1 hour at 37°C. Slides were mounted in ProLong Gold Antifade Mountant with DAPI (Thermo Fischer Scientific) and analyzed by confocal microscopy.

## Image acquisition and analysis

XYZ images were sampled according to Nyquist criterion using a Leica Leica STELLARIS5 confocal microscope platform, HC PL Apo objective (633, N.A.1.40), 405 nm diode/50 mW DMOD Flexibl, and 488, 555 and 647 laser lines in 470– 670 nm 80 MHz pulse continuum WLL2. Images were restored using a classic maximum likelihood restoration algorithm in the Huygens Professional Software (SVI, Hilversum, The Netherlands).^10^ The colocalization maps employing single pixel overlap coefficient values ranging from 0-1, were created in the Huygens Professional Software.^11^ The resulting overlap coefficient values are presented as the pseudo color which scale is shown in corresponding lookup tables (LUT).

# References

1. Baresova V, Krijt M, Skopova V, Souckova O, Kmoch S, Zikanova M. CRISPR-Cas9 induced mutations along de novo purine synthesis in HeLa cells result in accumulation of individual enzyme substrates and affect purinosome formation. *Mol Genet Metab*. 2016;119(3):270-7.

2. Pelet A, Skopova V, Steuerwald U, et al. PAICS deficiency, a new defect of de novo purine synthesis resulting in multiple congenital anomalies and fatal outcome. *Hum Mol Genet*. 2019;28(22):3805-14.

3. Souckova O, Skopova V, Baresova V, et al. Metabolites of De Novo Purine Synthesis: Metabolic Regulators and Cytotoxic Compounds. *Metabolites*. 2022;12(12).

4. Baresova V, Skopova V, Sikora J, et al. Mutations of ATIC and ADSL affect purinosome assembly in cultured skin fibroblasts from patients with AICA-ribosiduria and ADSL deficiency. *Hum Mol Genet*. 2012;21(7):1534-43.

5. Kmoch S, Hartmannova H, Stiburkova B, Krijt J, Zikanova M, Sebesta I. Human adenylosuccinate lyase (ADSL), cloning and characterization of full-length cDNA and its isoform, gene structure and molecular basis for ADSL deficiency in six patients. *Hum Mol Genet*. 2000;9(10):1501-13.

6. Krijt M, Souckova O, Baresova V, Skopova V, Zikanova M. Metabolic tools for identification of new mutations of enzymes engaged in purine synthesis leading to neurological impairment. *Folia Biol (Praha)*. 2019;65:152-7.

7. Weng WC, Skopova V, Baresova V, et al. Expanding clinical spectrum of PAICS deficiency: Comprehensive analysis of two sibling cases. *Eur J Hum Genet*. 2024.

8. Baresova V, Skopova V, Souckova O, Krijt M, Kmoch S, Zikanova M. Study of purinosome assembly in cell-based model systems with de novo purine synthesis and salvage pathway deficiencies. *PLoS One*. 2018;13(7):e0201432.

9. Madrova L, Krijt M, Baresova V, et al. Mass spectrometric analysis of purine de novo biosynthesis intermediates. *PLoS One*. 2018;13(12):e0208947.

10. Landmann L. Deconvolution improves colocalization analysis of multiple fluorochromes in 3D confocal data sets more than filtering techniques. *J Microsc*. 2002;208(Pt 2):134-47.

11. Manders EMM, Verbeek FJ, Aten JA. Measurement of co-localization of objects in dual-colour confocal images. *J Microsc*. 1993;169(3):375-82.
